# Triphenyl­bis[4-(trifluoro­meth­yl)benzoato-κ*O*]anti­mony(V)

**DOI:** 10.1107/S1600536809017449

**Published:** 2009-05-20

**Authors:** Li Quan, Handong Yin, Liansheng Cui, Minglei Yang, Daqi Wang

**Affiliations:** a College of Chemistry and Chemical Engineering, Liaocheng University, Shandong 252059, People’s Republic of China

## Abstract

The title complex, [Sb(C_6_H_5_)_3_(C_8_H_4_F_3_O_2_)_2_], is located on a twofold axis defined by the metal center and two C atoms of a coordinated phenyl group. The environment of the Sb atom approximates a trigonal-bipyramidal geometry, with the axial positions occupied by the O atoms of symmetry-related 4-(trifluoro­meth­yl)benzoate ligands. In this ligand, the CF_3_ group is disordered by rotation about the C—C bond and the F atoms are distributed over two sets of sites with occupancies of 0.62 (3) and 0.38 (3). In the crystal, mol­ecules are assembled in a three-dimensional framework through weak C—H⋯O hydrogen bonds.

## Related literature

For related Sb(V) structures, see: Sharutin *et al.* (2003 ▶); Yin *et al.* (2008 ▶); Yu *et al.* (2004 ▶).
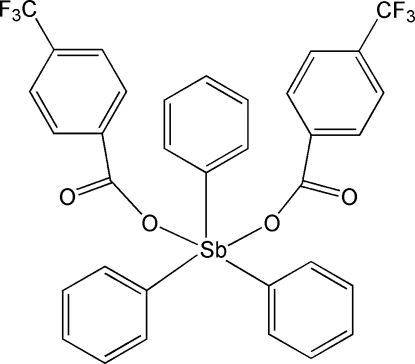


## Experimental

### 

#### Crystal data

[Sb(C_6_H_5_)_3_(C_8_H_4_F_3_O_2_)_2_]
*M* *_r_* = 731.27
Hexagonal, 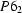

*a* = 12.9879 (10) Å
*c* = 16.042 (2) Å
*V* = 2343.5 (4) Å^3^
*Z* = 3
Mo *K*α radiation
μ = 0.96 mm^−1^
*T* = 298 K
0.44 × 0.31 × 0.24 mm

#### Data collection

Bruker SMART diffractometer
Absorption correction: multi-scan (*SADABS*; Sheldrick, 1996 ▶) *T*_min_ = 0.679, *T*_max_ = 0.803
9708 measured reflections
2719 independent reflections
1962 reflections with *I* > 2σ(*I*)
*R*_int_ = 0.053

#### Refinement

*R*[*F* ^2^ > 2σ(*F* ^2^)] = 0.058
*wR*(*F* ^2^) = 0.145
*S* = 1.08
2719 reflections
233 parameters
55 restraints
H-atom parameters constrained
Δρ_max_ = 0.90 e Å^−3^
Δρ_min_ = −0.41 e Å^−3^
Absolute structure: Flack (1983 ▶), 1284 Friedel pairs
Flack parameter: 0.04 (7)

### 

Data collection: *SMART* (Siemens, 1996 ▶); cell refinement: *SAINT* (Siemens, 1996 ▶); data reduction: *SAINT*; program(s) used to solve structure: *SHELXS97* (Sheldrick, 2008 ▶); program(s) used to refine structure: *SHELXL97* (Sheldrick, 2008 ▶); molecular graphics: *ORTEP-3* (Farrugia, 1997 ▶) and *DIAMOND* (Brandenburg, 1998 ▶); software used to prepare material for publication: *SHELXTL* (Sheldrick, 2008 ▶).

## Supplementary Material

Crystal structure: contains datablocks I, global. DOI: 10.1107/S1600536809017449/bh2229sup1.cif

Structure factors: contains datablocks I. DOI: 10.1107/S1600536809017449/bh2229Isup2.hkl

Additional supplementary materials:  crystallographic information; 3D view; checkCIF report

## Figures and Tables

**Table:** 

Sb1—C9	2.087 (10)
Sb1—C15	2.103 (10)
Sb1—O1	2.150 (5)

**Table:** 

C9—Sb1—C9^i^	140.0 (5)
C9—Sb1—C15	110.0 (3)
O1—Sb1—O1^i^	176.0 (3)

Symmetry code: (i) 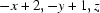.

**Table 2:** Hydrogen-bond geometry (Å, °)

*D*—H⋯*A*	*D*—H	H⋯*A*	*D*⋯*A*	*D*—H⋯*A*
C3—H3⋯O2^ii^	0.93	2.55	3.304 (13)	138

Symmetry code: (ii) 
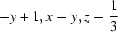
.

**Fig. 1.**
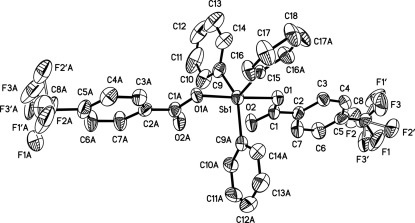
The molecular structure of the title complex, with atom labels and 50% probability displacement ellipsoids for non-H atoms.

**Fig. 2.**
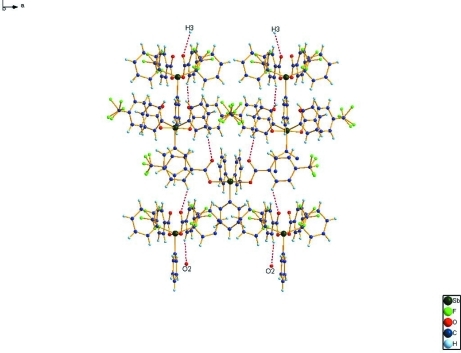
A chain of molecules linked by C3—H3···O2#1 intermolecular hydrogen bonds (dashed lines) (symmetry code #1: 1 - y, x-y, z - 1/3).

## supplementary crystallographic information

### Comment

Some triorganoantimony(V) complexes with acetylferroceneoxime as ligand showed *in vitro* antitumor activity (Yin *et al.*, 2008). The related title compound may show similar activity. The title complex is also related to triphenyl-bis(4-methylbenzoato-*κO*)-antimony(V), previously characterized (Sharutin *et al.*, 2003), although both complexes are not isostructural and crystallize in different space groups.

The crystal structure of the title complex consists of isolated molecules which have *C*2 molecular symmetry. The 2-fold axis is defined by atoms Sb1/C15/C18. The coordination geometry around the antimony center is best described as a distorted trigonal bipyramid. Two carboxylate groups occupy the axial sites with O1—Sb1—-O1^i^ angle being 176.0 (3)° [symmetry code: (i) 2 - *x*, 1 - *y*, *z*]. In the equatorial plane, the sum of angles C9—Sb1—C9^i^, C9—Sb1—C15 and C15—Sb1—C9^i^ is 360.0°. The Sb1—O1 bond length, 2.150 (5) Å, is significantly different from the corresponding distance in [4-(C_5_H_5_FeC_5_H_4_)C_6_H_4_COO]_2_Sb(C_6_H_4_F-4)_3_, 2.087 (6) Å (Yu *et al.*, 2004), but much shorter than the sum of the van der Waals radii for Sb and O, 3.2 Å. The Sb—C distances fall in the expected range found in the literature (Yu *et al.*, 2004).

### Experimental

4-Trifluoromethylbenzoic acid (0.152 g, 0.8 mmol) and sodium methoxide (0.8 mmol) were added to a stirring solution containing dichlorotriphenylantimony (0.172 g, 0.4 mmol) in toluene (25 ml). After refluxing for 8 h., a colorless solution was obtained and then filtered. The solvent was gradually removed by evaporation under vacuum until a white solid was obtained. The solid was recrystallized from petroleum ether/dichoromethane (1:1) to give colorless crystals of the title complex.

### Refinement

H atoms were placed in calculated positions and refined as riding atoms with C—H = 0.93 Å and *U*_iso_(H) = 1.2*U*_eq_(carrier C). F atoms were found to be disordered over two positions: F1/F1', F2/F2' and F3/F3'. Their occupancies were refined with the sum constrained to unity, and converged to 0.62 (3) [F1/F2/F3] and 0.38 (3) [F1'/F2'/F3']. Geometry was restrained (restraints not given). The Flack parameter has been refined (1284 measured Friedel pairs), although not documented by authors.

### Crystal data

**Table:**

[Sb(C_6_H_5_)_3_(C_8_H_4_F_3_O_2_)_2_]	*D*_x_ = 1.554 Mg m^−^^3^
*M**_r_* = 731.27	Mo *K*α radiation, λ = 0.71073 Å
Hexagonal, *P*6_2_	Cell parameters from 3513 reflections
Hall symbol: P 62	θ = 2.2–23.0°
*a* = 12.9879 (10) Å	µ = 0.96 mm^−^^1^
*c* = 16.042 (2) Å	*T* = 298 K
*V* = 2343.5 (4) Å^3^	Block, colorless
*Z* = 3	0.44 × 0.31 × 0.24 mm
*F*(000) = 1092	

### Data collection

**Table:**

Bruker SMART diffractometer	2719 independent reflections
Radiation source: fine-focus sealed tube	1962 reflections with *I* > 2σ(*I*)
graphite	*R*_int_ = 0.053
φ and ω scans	θ_max_ = 25.0°, θ_min_ = 1.8°
Absorption correction: multi-scan (*SADABS*; Sheldrick, 1996)	*h* = −15→9
*T*_min_ = 0.679, *T*_max_ = 0.803	*k* = −15→12
9708 measured reflections	*l* = −19→18

### Refinement

**Table:**

Refinement on *F*^2^	Secondary atom site location: difference Fourier map
Least-squares matrix: full	Hydrogen site location: inferred from neighbouring sites
*R*[*F*^2^ > 2σ(*F*^2^)] = 0.058	H-atom parameters constrained
*wR*(*F*^2^) = 0.145	*w* = 1/[σ^2^(*F*_o_^2^) + (0.0684*P*)^2^ + 2.131*P*] where *P* = (*F*_o_^2^ + 2*F*_c_^2^)/3
*S* = 1.08	(Δ/σ)_max_ = 0.026
2719 reflections	Δρ_max_ = 0.90 e Å^−^^3^
233 parameters	Δρ_min_ = −0.41 e Å^−^^3^
55 restraints	Absolute structure: Flack (1983), 1284 Friedel pairs
Primary atom site location: structure-invariant direct methods	Flack parameter: 0.04 (7)

### Fractional atomic coordinates and isotropic or equivalent isotropic displacement parameters (Å^2^)

**Table:**

	*x*	*y*	*z*	*U*_iso_*/*U*_eq_	Occ. (<1)
Sb1	1.0000	0.5000	0.10910 (15)	0.0641 (3)	
F1	0.2113 (17)	0.1590 (11)	0.182 (2)	0.26 (2)	0.62 (3)
F2	0.241 (2)	0.294 (3)	0.2756 (10)	0.25 (2)	0.62 (3)
F3	0.2382 (14)	0.3226 (13)	0.1459 (10)	0.127 (8)	0.62 (3)
F1'	0.242 (3)	0.3496 (19)	0.228 (3)	0.23 (3)	0.38 (3)
F2'	0.209 (3)	0.215 (3)	0.1356 (11)	0.24 (3)	0.38 (3)
F3'	0.2233 (18)	0.1848 (16)	0.2607 (10)	0.096 (9)	0.38 (3)
O1	0.8139 (5)	0.4442 (5)	0.1045 (4)	0.0653 (16)	
O2	0.8310 (6)	0.4481 (7)	0.2417 (4)	0.092 (2)	
C1	0.7700 (8)	0.4310 (9)	0.1798 (6)	0.070 (3)	
C2	0.6436 (8)	0.3930 (8)	0.1835 (6)	0.065 (2)	
C3	0.5766 (9)	0.3813 (9)	0.1133 (7)	0.074 (3)	
H3	0.6129	0.4011	0.0612	0.089*	
C4	0.4582 (10)	0.3412 (10)	0.1200 (9)	0.092 (3)	
H4	0.4137	0.3321	0.0723	0.110*	
C5	0.4034 (10)	0.3138 (10)	0.1967 (9)	0.088 (3)	
C6	0.4660 (10)	0.3246 (12)	0.2652 (8)	0.102 (4)	
H6	0.4287	0.3057	0.3170	0.122*	
C7	0.5843 (9)	0.3631 (12)	0.2599 (7)	0.098 (4)	
H7	0.6266	0.3697	0.3082	0.117*	
C8	0.2710 (11)	0.2707 (11)	0.2030 (8)	0.136 (7)	
C9	1.0390 (9)	0.6667 (9)	0.1536 (6)	0.077 (3)	
C10	1.0489 (11)	0.6986 (11)	0.2367 (6)	0.108 (4)	
H10	1.0400	0.6439	0.2776	0.130*	
C11	1.0718 (14)	0.8097 (13)	0.2596 (12)	0.157 (9)	
H11	1.0778	0.8304	0.3156	0.189*	
C12	1.0856 (16)	0.8904 (16)	0.1985 (12)	0.159 (9)	
H12	1.1002	0.9657	0.2134	0.191*	
C13	1.0781 (13)	0.8602 (11)	0.1153 (12)	0.146 (6)	
H13	1.0888	0.9154	0.0744	0.176*	
C14	1.0547 (11)	0.7483 (10)	0.0928 (8)	0.102 (4)	
H14	1.0496	0.7278	0.0368	0.122*	
C15	1.0000	0.5000	−0.0220 (6)	0.053 (3)	
C16	1.0867 (9)	0.4904 (10)	−0.0660 (7)	0.090 (3)	
H16	1.1470	0.4863	−0.0377	0.108*	
C17	1.0832 (12)	0.4871 (14)	−0.1517 (7)	0.120 (5)	
H17	1.1387	0.4757	−0.1807	0.144*	
C18	1.0000	0.5000	−0.1948 (9)	0.116 (7)	
H18	1.0000	0.5000	−0.2528	0.139*	

### Atomic displacement parameters (Å^2^)

**Table:**

	*U*^11^	*U*^22^	*U*^33^	*U*^12^	*U*^13^	*U*^23^
Sb1	0.0578 (5)	0.0795 (7)	0.0433 (3)	0.0256 (5)	0.000	0.000
F1	0.076 (11)	0.068 (10)	0.60 (7)	0.003 (8)	−0.06 (3)	0.05 (2)
F2	0.15 (2)	0.44 (6)	0.22 (2)	0.19 (3)	0.083 (19)	0.13 (3)
F3	0.065 (9)	0.100 (11)	0.22 (2)	0.043 (8)	−0.020 (10)	−0.016 (11)
F1'	0.11 (3)	0.083 (18)	0.50 (9)	0.047 (16)	0.09 (5)	0.03 (4)
F2'	0.074 (17)	0.37 (7)	0.20 (4)	0.06 (3)	−0.015 (17)	0.15 (4)
F3'	0.053 (11)	0.092 (16)	0.109 (16)	0.011 (10)	0.013 (10)	−0.009 (11)
O1	0.053 (3)	0.088 (4)	0.048 (3)	0.030 (3)	0.003 (3)	−0.003 (3)
O2	0.051 (4)	0.144 (7)	0.054 (4)	0.029 (4)	−0.005 (3)	0.000 (4)
C1	0.053 (6)	0.086 (7)	0.057 (5)	0.024 (5)	−0.002 (4)	−0.005 (4)
C2	0.058 (6)	0.060 (6)	0.062 (5)	0.019 (5)	0.000 (4)	−0.004 (4)
C3	0.068 (6)	0.084 (7)	0.062 (5)	0.033 (5)	−0.003 (6)	0.003 (6)
C4	0.075 (7)	0.093 (8)	0.105 (9)	0.040 (6)	−0.027 (7)	0.010 (7)
C5	0.060 (7)	0.071 (7)	0.121 (10)	0.024 (6)	0.014 (7)	0.024 (7)
C6	0.068 (7)	0.141 (11)	0.077 (8)	0.037 (7)	0.017 (6)	0.020 (8)
C7	0.069 (7)	0.154 (12)	0.054 (6)	0.044 (7)	0.004 (5)	0.000 (6)
C8	0.068 (10)	0.116 (15)	0.21 (2)	0.032 (10)	0.009 (12)	0.062 (14)
C9	0.055 (6)	0.082 (7)	0.085 (7)	0.027 (6)	0.013 (5)	−0.002 (6)
C10	0.086 (8)	0.109 (10)	0.100 (9)	0.026 (7)	0.016 (7)	−0.040 (7)
C11	0.097 (11)	0.147 (16)	0.17 (2)	0.019 (11)	0.027 (12)	−0.087 (15)
C12	0.108 (13)	0.096 (13)	0.23 (3)	0.016 (11)	0.036 (15)	−0.056 (14)
C13	0.107 (11)	0.098 (11)	0.21 (2)	0.030 (9)	0.033 (14)	0.001 (13)
C14	0.097 (9)	0.074 (8)	0.118 (11)	0.031 (7)	0.015 (8)	−0.007 (8)
C15	0.048 (7)	0.067 (8)	0.032 (5)	0.019 (6)	0.000	0.000
C16	0.079 (7)	0.141 (9)	0.056 (6)	0.060 (7)	−0.012 (5)	−0.019 (7)
C17	0.115 (10)	0.216 (16)	0.061 (7)	0.106 (11)	−0.005 (6)	−0.034 (8)
C18	0.097 (13)	0.22 (2)	0.037 (7)	0.083 (14)	0.000	0.000

### Geometric parameters (Å, °)

**Table:**

Sb1—C9	2.087 (10)	C6—C7	1.359 (15)
Sb1—C9^i^	2.087 (10)	C6—H6	0.9300
Sb1—C15	2.103 (10)	C7—H7	0.9300
Sb1—O1	2.150 (5)	C9—C14	1.377 (9)
Sb1—O1^i^	2.150 (5)	C9—C10	1.383 (9)
F1—C8	1.302 (9)	C10—C11	1.370 (9)
F2—C8	1.310 (10)	C10—H10	0.9300
F3—C8	1.328 (9)	C11—C12	1.380 (10)
F1'—C8	1.317 (10)	C11—H11	0.9300
F2'—C8	1.323 (10)	C12—C13	1.380 (10)
F3'—C8	1.341 (10)	C12—H12	0.9300
O1—C1	1.311 (11)	C13—C14	1.376 (9)
O2—C1	1.219 (10)	C13—H13	0.9300
C1—C2	1.459 (13)	C14—H14	0.9300
C2—C3	1.386 (14)	C15—C16^i^	1.386 (12)
C2—C7	1.395 (13)	C15—C16	1.386 (12)
C3—C4	1.359 (14)	C16—C17	1.375 (16)
C3—H3	0.9300	C16—H16	0.9300
C4—C5	1.375 (18)	C17—C18	1.362 (16)
C4—H4	0.9300	C17—H17	0.9300
C5—C6	1.333 (17)	C18—C17^i^	1.362 (16)
C5—C8	1.522 (16)	C18—H18	0.9300
			
C9—Sb1—C9^i^	140.0 (5)	F2—C8—F2'	133 (2)
C9—Sb1—C15	110.0 (3)	F1'—C8—F2'	110.0 (16)
C9^i^—Sb1—C15	110.0 (3)	F3'—C8—F2'	102.1 (13)
C9—Sb1—O1	90.6 (3)	F1—C8—F2'	47.2 (14)
C9^i^—Sb1—O1	90.8 (3)	F3—C8—F2'	56.8 (15)
C15—Sb1—O1	88.02 (16)	F2—C8—C5	112.6 (15)
C9—Sb1—O1^i^	90.8 (3)	F1'—C8—C5	116.3 (18)
C9^i^—Sb1—O1^i^	90.6 (3)	F3'—C8—C5	108.6 (13)
C15—Sb1—O1^i^	88.02 (16)	F1—C8—C5	109.0 (13)
O1—Sb1—O1^i^	176.0 (3)	F3—C8—C5	111.0 (11)
C1—O1—Sb1	110.8 (6)	F2'—C8—C5	114.2 (18)
O2—C1—O1	121.7 (9)	C14—C9—C10	119.7 (11)
O2—C1—C2	123.2 (8)	C14—C9—Sb1	115.0 (7)
O1—C1—C2	115.1 (8)	C10—C9—Sb1	125.3 (9)
C3—C2—C7	117.0 (9)	C11—C10—C9	120.8 (13)
C3—C2—C1	122.8 (9)	C11—C10—H10	119.6
C7—C2—C1	120.1 (9)	C9—C10—H10	119.6
C4—C3—C2	120.4 (11)	C10—C11—C12	119.1 (17)
C4—C3—H3	119.8	C10—C11—H11	120.4
C2—C3—H3	119.8	C12—C11—H11	120.4
C3—C4—C5	120.7 (11)	C13—C12—C11	120.5 (18)
C3—C4—H4	119.7	C13—C12—H12	119.8
C5—C4—H4	119.7	C11—C12—H12	119.8
C6—C5—C4	120.1 (11)	C14—C13—C12	120.0 (16)
C6—C5—C8	120.1 (12)	C14—C13—H13	120.0
C4—C5—C8	119.8 (12)	C12—C13—H13	120.0
C5—C6—C7	120.2 (11)	C9—C14—C13	119.8 (13)
C5—C6—H6	119.9	C9—C14—H14	120.1
C7—C6—H6	119.9	C13—C14—H14	120.1
C6—C7—C2	121.6 (11)	C16^i^—C15—C16	118.8 (12)
C6—C7—H7	119.2	C16^i^—C15—Sb1	120.6 (6)
C2—C7—H7	119.2	C16—C15—Sb1	120.6 (6)
F2—C8—F1'	46.9 (16)	C17—C16—C15	119.7 (10)
F2—C8—F3'	60.8 (14)	C17—C16—H16	120.1
F1'—C8—F3'	104.1 (14)	C15—C16—H16	120.1
F2—C8—F1	114.2 (14)	C18—C17—C16	121.3 (11)
F1'—C8—F1	135 (2)	C18—C17—H17	119.3
F3'—C8—F1	58.8 (14)	C16—C17—H17	119.3
F2—C8—F3	106.8 (13)	C17—C18—C17^i^	118.9 (15)
F1'—C8—F3	61.8 (19)	C17—C18—H18	120.5
F3'—C8—F3	140.1 (15)	C17^i^—C18—H18	120.5
F1—C8—F3	102.8 (12)		

Symmetry codes: (i) −*x*+2, −*y*+1, *z*.

### Hydrogen-bond geometry (Å, °)

**Table:**

*D*—H···*A*	*D*—H	H···*A*	*D*···*A*	*D*—H···*A*
C3—H3···O2^ii^	0.93	2.55	3.304 (13)	138

Symmetry codes: (ii) −*y*+1, *x*−*y*, *z*−1/3.
